# Sophoridine attenuates osteoarthritis progression: association with suppression of chondrocyte pyroptosis via inhibiting NF-κB signaling pathway

**DOI:** 10.3389/fphar.2026.1861567

**Published:** 2026-07-15

**Authors:** Ludan Zhang, Xinyu Xing, Chengcong Zhou, Fangda Fu, Zikun Chen, Chengliang Wu, Hongfeng Ruan, Ming Yue

**Affiliations:** 1 Department of Physiology, College of Basic Medical Sciences, Zhejiang Chinese Medical University, Hangzhou, Zhejiang, China; 2 Institute of Orthopaedic and Traumatology, The First Affiliated Hospital of Zhejiang Chinese Medical University (Zhejiang Provincial Hospital of Traditional Chinese Medicine), Hangzhou, Zhejiang, China; 3 Key Laboratory of Blood-stasis-toxin Syndrome of Zhejiang Province, Zhejiang Chinese Medical University, Hangzhou, Zhejiang, China

**Keywords:** inflammation, NF-κB, osteoarthritis, pyroptosis, sophoridine

## Abstract

**Introduction:**

Osteoarthritis (OA) is a degenerative musculoskeletal disease characterized by cartilage degradation and inflammation. Sophoridine (SR), a quinolizidine alkaloid from traditional Chinese herbs, has demonstrated anti-inflammatory properties, but its role in OA is unknown.

**Methods:**

The effects of SR on chondrocyte viability were assessed using the Cell Counting Kit-8 (CCK-8) assay. Interleukin-1β (IL-1β)-stimulated murine primary chondrocytes were used as an *in vitro* OA model. Extracellular matrix (ECM) synthesis was evaluated by toluidine blue staining. Key molecules involved in ECM metabolism, inflammation, and pyroptosis, as well as nuclear factor kappa B (NF-κB) signaling activity, were analyzed using quantitative reverse transcription PCR (RT-qPCR), Western blotting, and immunofluorescence. Chondrocyte morphological changes were examined by scanning electron microscopy. To validate causal mechanisms, a functional rescue experiment was performed *in vitro* using a specific NF-κB activator. For *in vivo* studies, an anterior cruciate ligament transection (ACLT)-induced murine OA model was established to evaluate the therapeutic efficacy of SR. OA progression was assessed by functional tests, micro-computed tomography (micro-CT), and histopathological analyses. Immunohistochemistry and immunofluorescence were further performed to evaluate molecular and signaling alterations in cartilage tissues.

**Results:**

In IL-1β-stimulated chondrocytes, SR (20 and 40 μg/mL) significantly inhibited ECM degradation and suppressed the expression of pro-inflammatory cytokines. *In vivo* studies utilizing a well-established murine OA model induced by ACLT demonstrated that intraperitoneal SR administration (7.5 and 15 mg/kg/day for 8 weeks) markedly ameliorated cartilage destruction and inflammation. Notably, SR prominently reduced chondrocyte pyroptosis and suppressed NF-κB pathway activation. Mechanistically, co-treatment with an NF-κB activator significantly reversed the SR-mediated protection of ECM components and abolished the downregulation of key pyroptosis-related proteins.

**Conclusion:**

Our study indicates that SR effectively attenuates OA progression, which was associated with the suppression of chondrocyte pyroptosis and inflammation via NF-κB signaling pathway inhibition, suggesting SR as a promising therapeutic agent for OA treatment.

## 1 Introduction

Osteoarthritis (OA) represents a chronic degenerative joint disorder predominantly affecting the middle-aged and elderly populations, marked by progressive cartilage degradation, synovial inflammation, and subchondral bone remodeling. With the global population aging and working lives extending, the incidence of OA has been increasing annually. By 2020, an estimated 595 million individuals worldwide were affected by OA, representing 7.6% of the global population, and imposing a substantial burden on individuals, healthcare systems, and economies (Collaborators, 2023). Despite this significant disease burden, current therapies for OA remain largely palliative, primarily focusing on symptom relief while lacking approved disease-modifying agents that reverse disease progression. This critical therapeutic gap underscores the urgent need for novel therapeutic strategies that target the underlying pathophysiological mechanisms of disease pathogenesis (Glinkowski and Tomaszewski, 2025).

Cartilage degeneration serves as the hallmark feature of OA and is largely attributed to the failure of chondrocytes to maintain homeostasis between extracellular matrix (ECM) synthesis and degradation (Sengprasert et al., 2023; McAllister et al., 2018). Mounting evidence indicates that chronic, low-grade inflammation plays a central role in disrupting this equilibrium. Pro-inflammatory cytokines, such as interleukin-1β (IL-1β) and tumor necrosis factor-α (TNFα), stimulate the expression of cartilage-degrading enzymes, including matrix metalloproteinases (MMPs) and a disintegrin and metalloproteinase with thrombospondin motifs (ADAMTSs), thereby accelerating ECM breakdown. Moreover, these cytokines establish a detrimental positive feedback loop by stimulating chondrocytes to produce additional inflammatory mediators, which further amplify local inflammation and accelerate tissue degradation (van den Bosch et al., 2024; Fang et al., 2024).

Pyroptosis, a highly inflammatory form of programmed cell death, has emerged as a critical driver of inflammation within the osteoarthritic joint and has been recognized as a key contributor to OA pathogenesis (Lin et al., 2025; An et al., 2020). The canonical pyroptosis is mediated by inflammasome assembly, accompanied by Caspase-1-mediated gasdermin D (GSDMD) cleavage and the subsequent release of IL-1β and IL-18 (Liu et al., 2024b; Yu et al., 2021b). Notably, this process has been observed in multiple joint-resident cell types, including chondrocytes, fibroblast-like synoviocytes (FLS), and macrophages, all of which contribute to the pro-inflammatory microenvironment that accelerates cartilage degradation and joint dysfunction (Lin et al., 2025; An et al., 2020). Given its central role in OA pathogenesis, pyroptosis has gained considerable attention as a promising therapeutic target in OA. Preclinical studies have demonstrated that inhibition of the NLR family pyrin domain containing 3 (NLRP3) inflammasome with specific inhibitors such as MCC950 and CY-09 effectively protects against cartilage degradation in destabilization of the medial meniscus (DMM)-induced mouse OA models (Ni et al., 2021; Zhang et al., 2021).

Nuclear factor-κB (NF-κB) functions as a pivotal mediator of inflammatory responses, and plays a pivotal role in the pathogenesis of OA (Liu et al., 2017; Choi et al., 2019). During the OA progression, aberrant NF-κB activation not only stimulates chondrocytes to produce pro-inflammatory factors such as IL-1β, IL-6, and tumor necrosis factor-α (TNF-α), but also upregulates matrix-degrading enzymes and other catabolic factors (Choi et al., 2019). Moreover, NF-κB has been identified as a key transcriptional regulator of pyroptosis, controlling the transcription of essential components of inflammasomes, such as NLRP3, and pro-IL-1β (Swanson et al., 2019; Zhong et al., 2016). Increasing evidence supports the NF-κB/NLRP3 signaling axis as a highly attractive therapeutic target in OA. Accordingly, growing research efforts have identified candidate compounds that alleviate OA by inhibiting NLRP3 inflammasome-mediated chondrocyte pyroptosis through suppression of NF-κB activation (Zhang et al., 2024; Wang et al., 2024; Zhou et al., 2024).

Sophoridine (SR) is a natural quinolizidine alkaloid predominantly found in several traditional Chinese herbs, such as Sophora alopecuroides L. and Euchresta japonica Benth (Tang et al., 2022; Wang et al., 2022). Extensive studies have demonstrated that SR possesses a wide range of biological activities, including anticancer, anti-inflammatory, antiviral, antiarrhythmic, and analgesic effects (Dai et al., 2021; Liang et al., 2022; Ren et al., 2019; Ur Rashid et al., 2020; Zhang et al., 2006). Of particular clinical relevance, SR hydrochloride injection, known for its high efficacy and low toxicity, was approved by the China Food and Drug Administration (CFDA) as an anticancer drug in 2005 (Ur Rashid et al., 2020). Regarding its anti-inflammatory potential, SR has been demonstrated to inhibit the expression of pro-inflammatory mediators including IL-1β, TNF-α, and IL-6, primarily through inhibition of the NF-κB pathway (Huang et al., 2014; Wang et al., 2025). Strikingly, recent evidence from a collagen-induced arthritis (CIA) rat model demonstrated that SR effectively alleviates synovitis and bone erosion by reducing IL-1β and TNF-α production through inhibition of NF-κB and mitogen-activated protein kinase (MAPK) pathways (Chen et al., 2024a). However, despite these promising anti-inflammatory properties, the therapeutic role of SR in OA remains unexplored. Given its well-established anti-inflammatory properties and demonstrated capacity to inhibit NF-κB signaling, SR holds significant promise as a potential disease-modifying agent for OA.

In the present study, we systematically assessed the chondroprotective effects of SR in mitigating ECM degradation and inflammation in IL-1β-stimulated chondrocytes, and evaluated its therapeutic ability to prevent cartilage degeneration in an anterior cruciate ligament transection (ACLT)-induced OA mouse model. Furthermore, we comprehensively investigated the molecular mechanisms underlying SR’s protective effects, focusing on inflammation-related signaling pathways and chondrocyte pyroptosis.

## 2 Materials and methods

### 2.1 Chemicals and reagents

SR (CAS No. 6882–68–4, purity 98.2%) was purchased from the China Institute for Food and Drug Control (NIFDC). PCR primers were synthesized by Sangon Biotech Co., Ltd. (Shanghai, China). TRIzol® reagent was sourced from Invitrogen. High-Capacity RNA-to-cDNA Master Mix and SYBR Green PCR Master Mix were purchased from Transgen Biotech (China). Phosphatase/Protease Inhibitor Mixture was obtained from Thermo Fisher Scientific (Carlsbad, CA, United States). Fluorescent secondary antibodies were provided by LI-COR (Lincoln, NE, United States). The nuclear stain 4′,6-diamidino-2-phenylindole (DAPI) was obtained from Sungene Biotech (Tianjin, China). Fetal bovine serum (FBS) and Dulbecco’s Modified Eagle Medium (DMEM) were purchased from Gibco (Grand Island, NY, United States). Unless otherwise specified, all other chemicals were obtained from Sigma-Aldrich (St. Louis, MO, United States).

Primary antibodies used in this study included: Aggrecan (Affinity, #DF7561, lot: 1K8322), Collagen type II (Ruiying, #RLT1022, lot: B2201), MMP3 (Ruiying, #RLT4465, lot: B6501), MMP13 (Affinity, #AF5355, lot: 89a2870), ADAMTS5 (Ruiying, #RLN8690, lot: B21RC21), inducible nitric oxide synthase (iNOS; Proteintech, #18985-1-AP, lot: 00125485), COX-2 (Proteintech, #66351-1-Ig, lot: 10021385), TNF-α (Ruiying, #RLM3472, lot: B7201), interleukin-6 (IL-6; Proteintech, #26404-1-AP, lot: 00141313), NLRP3 (HUABIO, #ET1610-93, lot: H651014041), ASC (Proteintech, #30641-1-AP, lot: 00141309), GSDMD (Affinity, #DF13758, lot: 87w7452), Caspase-1/P20 (Proteintech, #22915-1-AP, lot: 00145866), IL-1β (BIOSS, #bs-6319R, lot: BD10306456), IL-18 (BIOSS, #bs-0529R, lot: BD0853446), p-p65 (CST, #3033S, lot: 19), total p65 (CST, #8242S, lot: 16), p-IκBα (Affinity, #AF2002, lot: 50z6879), IκBα (Ruiying, #RLT2419, lot: B1902), Lamin B1 (Ruiying, #RLT2522, lot: B2149), β-actin (OriGene, #TA811000, lot: WC105), GAPDH (OriGene, #TA02519, lot: F004).

### 2.2 Primary murine chondrocyte isolation and treatments

Primary chondrocytes were isolated from knee joint cartilage of male C57BL/6 mice aged 5–7 days using established aseptic surgical procedures. Briefly, articular cartilage was dissected and rinsed two to three times with phosphate-buffered saline (PBS). Subsequently, the cartilage tissues were digested with 0.2% Type II Collagenase (Biosharp, Hefei, Anhui, China) at 37 °C for 4–6 h. Following enzymatic digestion, the tissue suspension was centrifuged at 1000 × g for 5 min. The supernatant was discarded, and the resulting cell pellet was resuspended in DMEM medium (Gibco, Life Technologies, Carlsbad, CA, United States) supplemented with 10% FBS, and 1% penicillin-streptomycin.

Isolated chondrocytes were seeded at a density of 1 × 10^5^ cells/mL in 10-cm culture dishes and maintained at 37 °C in a humidified atmosphere containing 5% CO_2_. Culture medium was refreshed every 4 days. Upon reaching 80%–90% confluence, cells were detached using 0.25% trypsin-EDTA (Gibco, Life Technologies, Carlsbad, CA, United States) and passaged. To maintain chondrocyte phenotype and minimize dedifferentiation, only passage 1-3 cells were used for all experiments. Primary mouse chondrocytes were pretreated with SR (20 or 40 μg/mL) for 12 h, and then stimulated with IL-1β (10 ng/mL, PeproTech, Cranbury, NJ, United States) for 2, 12, or 24 h to simulate inflammatory chondrocytes in osteoarthritis (Li et al., 2024). To verify upstream dependency, primary chondrocytes were stimulated with IL-1β (10 ng/mL) and treated with SR (40 μg/mL) in the presence or absence of a specific NF-κB activator (10 μg/mL, MedChemExpress, Monmouth Junction, NJ, United States).

### 2.3 Cell viability assay

The cytotoxicity of SR toward chondrocytes was assessed using the Cell Counting Kit-8 (CCK-8 assay kit, GlpBio, Montclair, CA, United States) according to the manufacturer’s instructions. Chondrocyte suspensions were seeded into 96-well plates at 3 × 10^3^ cells/well and preincubated for 24 h at 37 °C under 5% CO_2_. Cells were then treated with SR (0, 5, 10, 20, 40, 80, or 160 μg/mL) for 24 or 48 h, 10 μL of CCK-8 solution was added to each well and incubated for 1 h at 37 °C. Absorbance was measured at 450 nm using a microplate reader (Thermo Fisher Scientific, Waltham, MA, United States). Each experimental condition was replicated in at least three independent chondrocyte cultures to ensure reproducibility and reliability of the findings.

### 2.4 Toluidine blue staining

To precisely quantify ECM content within murine cartilage, a standardized experimental method was executed. Primary chondrocytes were first isolated via enzymatic digestion and suspended in a trypsin-containing solution. A 10 μL aliquot of this suspension was dispensed into individual wells and incubated at 37 °C for approximately 1 hour to enable cellular attachment. Subsequently, 0.5 mL of DMEM containing 10% FBS was introduced into each well, followed by a 24-h incubation period at 37 °C. During this incubation, IL-1β was administered alongside SR at concentrations of 20 or 40 μg/mL to the cultures. Finally, on culture days 7–9, cells were fixed with 4% paraformaldehyde for 30 min and then stained with 0.1% toluidine blue solution (Sigma-Aldrich, St. Louis, MO, United States) for 30 min at room temperature. After washing with distilled water, the stained samples were scanned using an Epson V600 Photo Scanner (Japan). ImageJ software was used to analyze the resulting staining intensity to evaluate ECM levels in the cartilage specimens.

### 2.5 Scanning electron microscopy (SEM)

Cells were fixed with 2.5% glutaraldehyde for 30 min, and rinsed three times with PBS. They were dehydrated through a graded ethanol series (30%, 50%, 70%, 95%, and 100%) and dried using tert-butanol. The samples were then mounted on metal stubs and further dried in a silica gel vacuum desiccator. They were sputter-coated with gold and examined using a Hitachi-SU8010 scanning electron microscope operated at 15 kV (Chen et al., 2019).

### 2.6 Western blot analysis

Total protein was extracted from chondrocytes and lysed in RIPA buffer (Beyotime, Shanghai, China) supplemented with 1% protease inhibitor cocktail. The lysates were incubated on ice for 30 min, followed by centrifugation at 12,000 rpm for 10 min at 4 °C to obtain the supernatant. Protein concentrations were determined using the Pierce™ BCA Protein Assay Kit (Thermo Fisher Scientific, Waltham, MA, United States). Equal amounts of protein (30 μg) were separated by SDS-PAGE and transferred onto a nitrocellulose membrane (Millipore, Billerica, United States). After blocking with 5% non-fat milk at room temperature for 1 h, membranes were incubated overnight at 4 °C with primary antibodies against IL-6 (1:1000), TNF-α (1:1000), iNOS (1:1000), COX-2 (1:1000), Collagen II (1:500), Aggrecan (1:1000), MMP3 (1:1000), MMP13 (1:1000), ADAMTS5 (1:1000), NLRP3 (1:1000), ASC (1:1000), Caspase-1/P20 (1:1000), GSDMD (1:1000), IL-1β (1:1000), IL-18 (1:1000), NF-κB p65 (1:1000), phospho-NF-κB p65 (1:1000), IκBα (1:1000), phospho- IκBα (1:1000), GAPDH (1:1000), β-actin (1:1000) and Lamin B1 (1:1000). Subsequently, membranes were then incubated with IRDye 680 or IRDye 800-conjugated secondary antibodies for 2 h at room temperature. Protein bands were visualized using the Odyssey infrared imaging system (LI-COR Biosciences, Lincoln, NE, United States). Western blot experiments were performed using three independent biological replicates. GAPDH, β-actin or LaminB1 served as loading controls, and band intensities was quantified using ImageJ software (National Institutes of Health, Bethesda, MD, United States). All data were further normalized to the control group (set as 1).

### 2.7 Immunofluorescence

Cell culture inserts were sterilized and placed into a 12-well plate. Chondrocytes were seeded onto the inserts and incubated with or without SR for 12 h, followed by incubation with or without IL-1β (10 ng/mL) for 24 h. The cells were fixed with 4% paraformaldehyde at room temperature for 20 min, rinsed three times with PBS, and permeabilized using 0.1% Triton X-100 for 5 min at room temperature. The inserts were transferred to a humidified chamber and blocked with 5% bovine serum albumin at 37 °C for 1 h to prevent non-specific binding. The cells on the inserts were then incubated overnight at 4 °C with primary antibodies against Aggrecan (1:200), ADAMTS5 (1:200), ASC (1: 200) and NF-κB p65 (1:200). After washing with PBS, cells were incubated with fluorophore-conjugated secondary antibodies (Sungene Biotech, Tianjin, China) in the dark for 30 min, followed by DAPI counterstaining. The images were acquired using a fluorescence microscope (Carl Zeiss, Göttingen, Germany). Quantitative histomorphometric analysis was performed in a blinded manner using Image-Pro Plus Software version 6.0 (Media Cybernetics Inc., Rockville, Maryland, United States).

### 2.8 RNA extraction and quantitative RT-PCR

Chondrocytes were stimulated with IL-1β (10 ng/mL) with or without SR (20 μg/mL, 40 μg/mL). Total RNA was extracted using TRIzol™ Reagent (Invitrogen), and RNA concentration was measured using a NanoDrop 2000 (Thermo Fisher Scientific, United States). Complementary DNA (cDNA) was synthesized using High-Capacity RNA-to-cDNA Master Mix (Transgen Biotech, China) according to the manufacturer’s instructions. qPCR was performed using SYBR Green PCR Master Mix (Transgen Biotech, China) on the QuantStudio™ 7 Flex system (Thermo Fisher Scientific, United States) under the following conditions: denaturation at 95 °C for 1 min; 40 cycles of 95 °C for 10 s and 60 °C for 30 s. The primer sequences are listed in Table 1 β-actin served as the internal control, and relative gene expression levels were calculated using the 2^−ΔΔCT^ method (Livak and Schmittgen, 2001).

**TABLE 1:** Primer sequences.

Gene	Primers (Forward/Reverse)
*β-actin*	5′-GGC​TGT​ATT​CCC​CTC​CAT​CG-3′
	5′-CCA​GTT​GGT​AAC​AAT​GCC​ATG​T-3′
*Mmp3*	5′-ACA​TGG​AGA​CTT​TGT​CCC​TTT​TG-3′
	5′-TTG​GCT​GAG​TGG​TAG​AGT​CCC-3′
*Mmp13*	5′-CTT​CTT​CTT​GTT​GAG​CTG​GAC​TC-3′
	5′-CTG​TGG​AGG​TCA​CTG​TAG​ACT-3′
*Adamts5*	5′-GGA​GCG​AGG​CCA​TTT​ACA​AC-3′
	5′-CGT​AGA​CAA​GGT​AGC​CCA​CTT​T-3′
*Aggrecan*	5′-CCT​GCT​ACT​TCA​TCG​ACC​CC-3′
	5′-AGA​TGC​TGT​TGA​CTC​GAA​CCT-3′
*Col2a1*	5′-GGG​AAT​GTC​CTC​TGC​GAT​GAC-3′
	5′-GAA​GGG​GAT​CTC​GGG​GTT​G-3′
*iNos*	5′-GTT​CTC​AGC​CCA​ACA​ATA​CAA​GA-3′
	5′-GTG​GAC​GGG​TCG​ATG​TCA​C-3′
*Cox-2*	5′-TCC​TCA​CAT​CCC​TGA​GAA​CC-3′
	5′-GTC​GCA​CAC​TCT​GTT​GTG​CT-3′
*Tnf-α*	5′-CCT​GTA​GCC​CAC​GTC​GTA​G-3′
	5′-GGG​AGT​AGA​CAA​GGT​ACA​ACC-3′
*Il-6*	5′-CCA​AGA​GGT​GAG​TGC​TTC​CC-3′
	5′-CTG​TTG​TTC​AGA​CTC​TCT​CCC​T-3′
*Nlrp3*	5′-ATT​ACC​CGC​CCG​AGA​AAG​G-3′
	5′-TCG​CAG​CAA​AGA​TCC​ACA​CAG-3′
*Gsdmd*	5′-CCA​TCG​GCC​TTT​GAG​AAA​GTG-3′
	5′-ACA​CAT​GAA​TAA​CGG​GGT​TTC​C-3′
*Caspase-1*	5′-CTT​GGA​GAC​ATC​CTG​TCA​GGG-3′
	5′-AGT​CAC​AAG​ACC​AGG​CAT​ATT​CT-3′
*Il-1β*	5′-GCA​ACT​GTT​CCT​GAA​CTC​AAC​T-3′
	5′-ATC​TTT​TGG​GGT​CCG​TCA​ACT-3′
*Il-18*	5′-GTG​AAC​CCC​AGA​CCA​GAC​TG-3′
	5′-CCT​GGA​ACA​CGT​TTC​TGA​AAG​A-3′

### 2.9 Establishment of mouse OA model and experimental design

Eight-week-old male C57BL/6 mice (20 ± 2 g, n = 24) were purchased from the Animal Center of the Chinese Academy of Sciences in Shanghai, China. All mice were housed in Zhejiang Chinese Medical University Laboratory Animal Research Center under specific pathogen-free conditions (23 °C ± 2 °C; 12-h/12-h light/dark cycle) with *ad libitum* access to food/water. A mouse model of OA was established by transecting the ACLT (Li et al., 2024; Jin et al., 2023). Mice were randomized assigned to four groups (n = 6 per group): Sham group (articular capsule incision); OA model (Vehicle, ACLT surgery); Low-dose SR group (ACLT + 7.5 mg/(kgday) SR); High-dose SR group (ACLT + 15 mg/(kgday) SR). The *in vivo* dosages of SR (7.5 and 15 mg/kg) were selected based on a previous study where ovariectomized (OVX) mice were treated with 15 mg/kg of SR for 4 weeks (Zhao et al., 2017). Given that our study extended the treatment duration to 8 weeks, the 15 mg/kg dose was maintained as the high-dose group, and a lower dose of 7.5 mg/kg was established to safely and comprehensively evaluate the protective effects of SR over this longer administration period. The sample size was determined through power analysis based on the expected effect size and the variability observed in the preliminary study. After 1 week of adaptation, if the mice were 8 weeks old and their weight was between 20 and 22 g, they were included. If the mice showed any signs of illness or injury before the start of the experiment, they were excluded. Starting from the third day post-surgery, SR or saline (vehicle control) was administered via intraperitoneal injection. Animals were monitored daily for signs of pain or distress. Eight weeks later, mice were euthanized via intraperitoneal injection of an overdose of sodium pentobarbital (150 mg/kg). Death was confirmed by verifying the absence of a heartbeat and the presence of fixed, dilated pupils. The allocation sequence was concealed during the allocation phase, and Ludan Zhang and Xinyu Xing employed blinding throughout the experiment, outcome assessment, and data analysis to minimize bias. No data points were excluded during the analysis. All animal experiments were conducted in accordance with the ARRIVE guidelines and approved by the Ethics Committee of Zhejiang Chinese Medical University (IACUC-20240513–05).

### 2.10 Sensory and motor function tests

Mechanical Sensitivity Assessment: After a 30-min habituation period, the central plantar region of the hindpaw was stimulated using a Von Frey filament until bending. A paw withdrawal within 1 s was recorded as positive response. Five stimulations were applied per trial; ≥3 positive responses scored “ × ” (otherwise “O”). Force levels were adjusted using the up-down method over six cycles. The last force eliciting “ × ” was documented.

Thermal Hyperalgesia Testing: Thermal withdrawal latency (TWL) was measured using a Hargreaves device. Following habituation, radiant heat was applied to the hindpaw, and time to withdrawal (lifting/licking/retraction) was recorded.

Gait analysis: Locomotion was assessed using DigiGait system. Mice were pre-trained on the treadmill. During testing (≥30-min acclimation; 18 cm/s speed), high-speed imaging was used to capture paw movements. The affected right/contralateral left hindlimb ratio (RH/LH) was calculated to normalize for individual variability (Qian et al., 2024).

Behavioral Testing Conditions: All assessments (mechanical/thermal sensitivity, gait analysis) were performed in identical temperature- and humidity-controlled chambers with elevated metal mesh flooring. All procedures were performed by investigators blinded to the experimental groups (Ge et al., 2024).

### 2.11 Micro computed tomography (Micro-CT) analysis

Prior to histological processing, knee joints were scanned using a high-resolution micro-CT system (Skyscan1176, Bruker micro-CT, Belgium) at 50 kV/500 μA with 9 μm resolution. Image reconstruction and morphometric analysis used NRecon v1.6 and CTAn v1.15, respectively. Three-dimensional images were reconstructed with CTVol v2.2 software. Coronal images of tibial subchondral bone were subjected to 3D histomorphometric analysis. Bone volume fraction (BV/TV), Trabecular thickness (Tb.Th), and trabecular separation (Tb.Sp) (1/mm) in the medial compartment were calculated from 50 consecutive images using XTek computational algorithms.

### 2.12 Histological staining

The knee joints were fixed in 4% paraformaldehyde for 24 h and decalcified with 14% EDTA solution for 4 weeks. Paraffin-embedded tissues were cut into 4-μm-thick sections and stained with hematoxylin-eosin (H&E) and Safranin O-Fast Green (Solarbio, Beijing, China). The degree of cartilage degeneration was assessed according to the OA Research Society International (OARSI) scoring system (Zhou et al., 2024).

### 2.13 Immunohistochemistry (IHC) and immunofluorescence (IF) analyses

For IHC, tissue sections were deparaffinized in xylene and rehydrated in gradient ethanol. The sections were incubated with 0.3% hydrogen peroxide and 5% bovine serum albumin at room temperature. Primary antibodies (Aggrecan, Collagen II, MMP3, MMP13, ADAMTS5, IL-6, TNF-α, iNOS, COX-2,NLRP3, GSDMD, Caspase-1, IL-1β, IL-18) were incubated overnight at 4 °C. The following day, sections were incubated with the polymer-HRP-labeled secondary antibody for 30 min. The immunohistochemical reaction was visualized by incubation with 0.05% diaminobenzidine (DAB). The positive cell staining rates were statistically analyzed with ImageJ. For the IF analysis, sections were incubated with primary antibodies, including ASC, p-p65, p65, overnight at 4 °C, then treated with fluorescently conjugated secondary antibody (Sungene Biotech, Tianjin, China) for 30 min in the dark. After DAPI counterstaining, the images were acquired using a fluorescence microscope (Carl Zeiss, Göttingen, Germany). Quantitative histomorphometric analysis was conducted in a blinded manner using Image-Pro Plus Software version 6.0 (Media Cybernetics Inc., Rockville, Maryland, United States) (Yu et al., 2021a).

### 2.14 Statistical analysis

For *in vitro* experiments, data were obtained from at least three independent biological replicates. For *in vivo* experiments, a sample size of n = 6 mice per group was utilized. All numerical data were presented as mean ± SD. One-way analysis of variance (ANOVA) and appropriate post-hoc comparisons were performed using GraphPad Prism 9 software (GraphPad Software Inc., La Jolla, CA). A *P* value of less than 0.05 was considered statistically significant. To confirm the statistical validity and reproducibility of the animal studies, post-hoc power calculations were performed specifically for the *in vivo* outcome measures using G*Power 3.1. For the animal experiments, these calculations confirmed that a sample size of n = 6 per group yielded a statistical power >0.89 for primary histological and structural outcomes, owing to high between-group effect sizes relative to tight intra-group variance. The calculated effect sizes, *F*-statistics, and exact achieved power values for each primary outcome measure are provided in Supplementary Table S1.

## 3 Results

### 3.1 Evaluation of SR on cytotoxicity of primary chondrocytes

**FIGURE 1:**
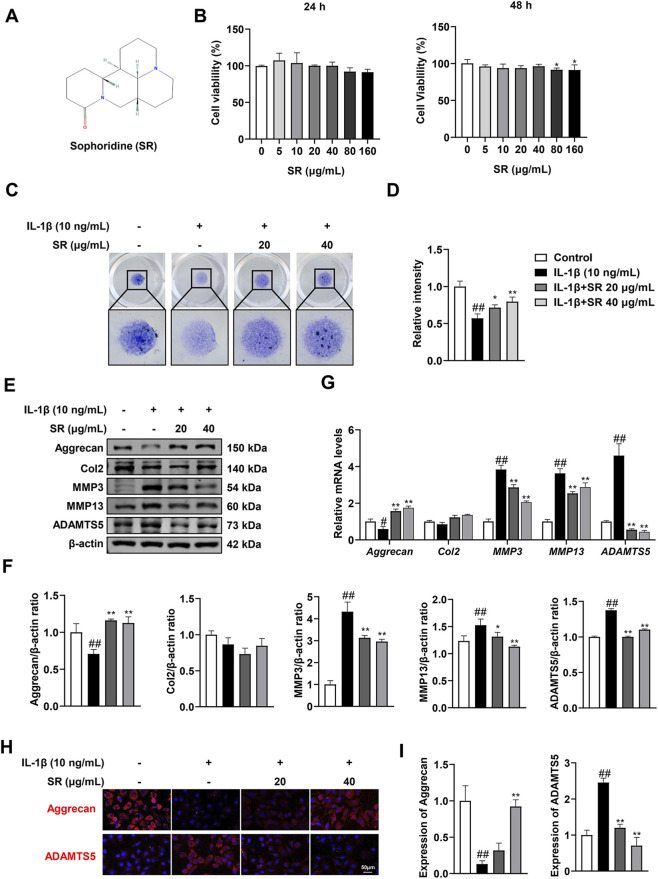
Sophoridine promotes extracellular matrix synthesis and inhibits catabolism. **(A)** Molecular structure of SR. **(B)** Cytotoxic effects of SR on the viability of mouse chondrocyte cells after treatment for 24 and 48 h. **(C)** Toluidine blue staining of high-density-cultured primary chondrocytes cells after treatment with different concentrations of SR and IL-1β. **(D)** The relative intensity of blue staining. **(E, F)** Western blotting results of extracellular matrix anabolic and catabolic related proteins Aggrecan,Col2, MMP3, MMP13, ADAMTS5 in chondrocytes quantified by ImageJ. **(G)** The expression levels of genes related to extracellular matrix synthesis and catabolism were quantified by RT-qPCR. **(H, I)** After IL-1β treatment with or without SR for 24 h, the expression of Aggrecan or ADAMTS5 was observed by immunofluorescence and the fluorescence intensity analyzed using Image **(J)**. DAPI stains nuclei blue. Data are presented as mean ± SD (n = 3 independent biological replicates). ^#^ *P* < 0.05, ^##^ *P* < 0.01, compared with the Control group; *P < 0.05, **P < 0.01, compared with the IL-1β group.

The chemical structure of SR is shown in Figure 1A. To assess its potential cytotoxicity, primary chondrocytes were exposed to increasing concentrations of SR (0, 5, 10, 20, 40, 80, and 160 μg/mL) for 24 or 48 h, and cell viability was subsequently evaluated using the CCK-8 assay. SR exhibited no significant cytotoxic effects at concentrations ≤40 μg/mL across both time points. However, a marked reduction in cell viability was observed at 80 and 160 μg/mL following 48 h of exposure (Figure 1B). To ensure maximal therapeutic efficacy while avoiding any off-target toxicity, SR concentrations of 20 and 40 μg/mL were selected as optimal doses for further *in vitro* studies.

### 3.2 SR exhibits protective effects on ECM homeostasis in IL-1β-stimulated chondrocytes

Effective modulation of cartilage ECM remodeling is essential for the prevention and treatment of OA, where chondrocytes play a pivotal role by regulating both ECM synthesis and degradation (Deng et al., 2022; Michelacci et al., 2023). To explore the protective potential of SR on ECM homeostasis under inflammatory conditions, we employed IL-1β-stimulated chondrocytes as an *in vitro* OA model. Toluidine blue staining was used to evaluate ECM synthesis in high-density chondrocyte cultures. IL-1β stimulation markedly reduced ECM content, as indicated by diminished staining intensity compared to the control group. Remarkably, SR treatment restored ECM production (Figures 1C,D), indicating a significant protective effect of SR against IL-1β-induced ECM loss.

To further characterize SR’s effects on ECM metabolism, we analyzed the expression of key anabolic and catabolic markers using Western blot and RT-qPCR analyses. IL-1β exposure significantly suppressed the expression of anabolic proteins, including aggrecan and collagen type II (Col2), while upregulating matrix-degrading enzymes MMP3, MMP13, and ADAMTS5. SR treatment effectively reversed these pathological changes with the exception of Col2 (Figures 1E–G), suggesting a beneficial shift toward ECM preservation. These findings were further validated by IF analysis, which confirmed increased aggrecan and decreased ADAMTS5 expression following SR intervention (Figures 1H,I). Together, these findings underscore the role of SR in maintaining ECM integrity under inflammatory stress.

### 3.3 SR effectively inhibits IL-1β-induced inflammatory response in chondrocytes

**FIGURE 2:**
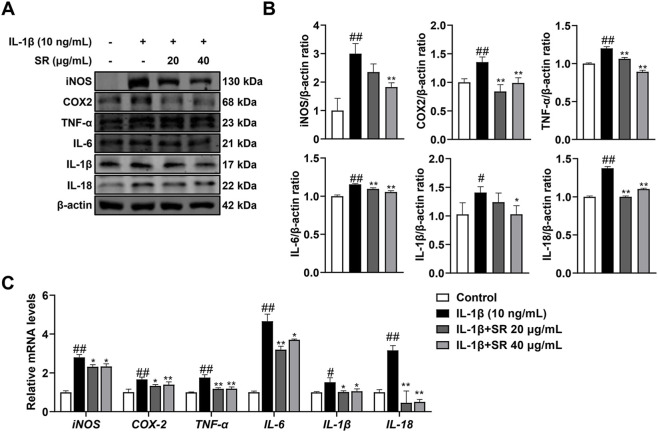
Anti-inflammation effects of SR in mouse chondrocytes. **(A-B)** The expression of iNOS, COX-2, TNF-α, IL-6, IL-1β, and IL-18 was visualized by Western blot and quantified using ImageJ. **(C)** The expression levels of inflammation-related genes were quantitatively analyzed by RT-qPCR method. n = 3. Data are presented as mean ± SD (n = 3 independent biological replicates). ^#^ *P* < 0.05, ^##^ *P* < 0.01, compared with the Control group; *P < 0.05, **P < 0.01, compared with the IL-1β group.

Inflammation is a major driver of ECM degradation and cartilage damage in OA (van den Bosch et al., 2024; Fang et al., 2024). To determine whether SR modulates inflammatory responses, we assessed the expression of pro-inflammatory mediators in IL-1β-stimulated chondrocytes, with or without SR treatment. IL-1β significantly upregulated the protein and mRNA levels of iNOS, COX-2, TNF-α, IL-6, IL-1β and IL-18. Notably, SR treatment effectively suppressed the upregulation of these inflammatory markers (Figure 2). These results demonstrate that SR exerts potent anti-inflammatory effects in chondrocytes under IL-1β stimulation, further supporting its therapeutic potential in OA.

### 3.4 SR alleviates OA progression in an ACLT-induced mouse model

**FIGURE 3:**
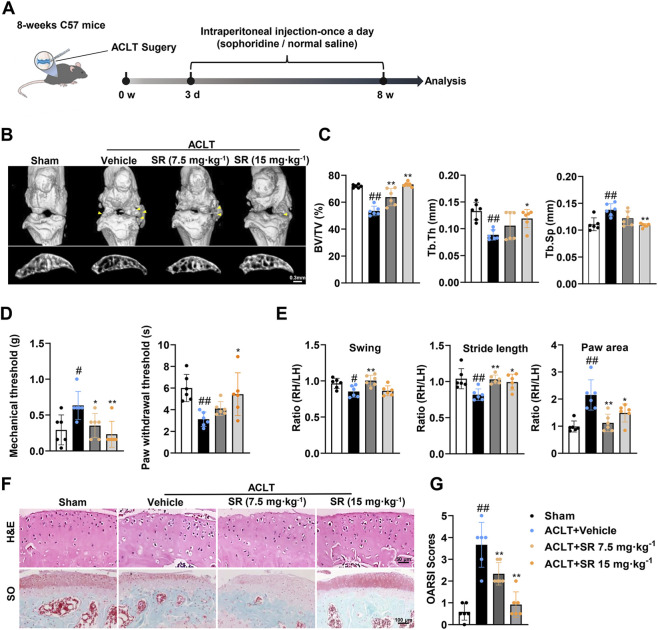
SR relieves OA development in a surgically induced mouse ACLT model. **(A)** ACLT surgery to establish OA model mice and treatment regimen. **(B)** Representative three-dimensional images of knee joints. Yellow triangles indicated osteophytes. **(C)** Statistical results of bone morphological parameters in **(B) (D–E)** Analysis of Mechanical Pain, Heat Pain, and Gait Alterations in Mice Following ACLT Modeling. **(F)** Sections of knee joints were stained with H&E and Safranin O-Fast Green. **(G)** OARSI scores of cartilage specimens of different groups. Data are presented as mean ± SD (n = 6 mice per group). ^#^ *P* < 0.05, ^##^ *P* < 0.01, compared with the Sham group; *P < 0.05, **P < 0.01, compared with the ACLT + Vehicle group.

To evaluate the *in vivo* therapeutic effect of SR for OA, we conducted an ACLT-induced OA mouse model. Mice were administered SR via intraperitoneal injection at two doses (7.5 and 15 mg/(kgday)) for 8 weeks following ACLT surgery. The detailed experimental scheme of ACLT induction and SR treatment is illustrated in Figure 3A joint reconstruction demonstrated that ACLT surgery led to prominent marginal osteophyte formation and narrowing of the joint space. SR treatment significantly preserved bone morphology, mitigating these degenerative changes. Similarly, 3D reconstruction of tibia subchondral bone showed that SR effectively prevented subchondral bone loss compared with the ACLT group (Figure 3B). Quantitative micro-CT analysis demonstrated that SR treatment significantly attenuated ACLT-induced subchondral bone degeneration with a significant effect at the higher dose, as evidenced by increased BV/TV and trabecular thickness (Tb.Th), along with reduced Tb. Sp (Figure 3C).

Functionally, SR, particularly at the high dose, effectively reduced pain hypersensitivity, as demonstrated by improvements in both mechanical and thermal pain thresholds in ACLT-operated mice (Figure 3D). In addition, gait analysis demonstrated that SR markedly improved ACLT-induced locomotor deficits, as reflected by restored stride length and improved hindlimb swing symmetry (Figure 3E).

Cartilage preservation was evaluated using H&E and Safranin O-Fast Green staining. In ACLT-induced mice, marked cartilage erosion and loss of proteoglycan staining were observed. In contrast, SR treatment—especially at the higher dose—attenuated these histological changes and preserved cartilage structure (Figure 3F). Correspondingly, OARSI scores were significantly higher in the ACLT group than in the sham group, whereas both the low and high doses of SR administration reduced these scores (Figure 3G). Taken together, these findings suggest that SR attenuates cartilage degeneration and ameliorates osteoarthritic phenotypes in this model.

### 3.5 SR attenuates cartilage matrix degradation and inflammation in OA mice

**FIGURE 4:**
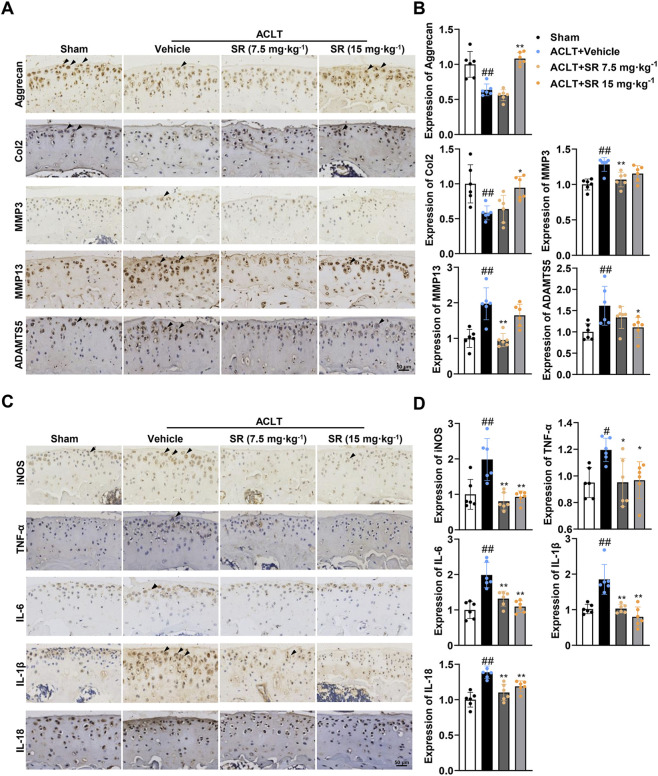
SR retards the degradation and inflammation of articular cartilage matrix. **(A)** Immunohistochemistry results of Aggrecan, Col2, MMP3, MMP13 and ADAMTS5 proteins. Black arrows indicate a strong positive expression in cartilage tissue. **(B)** The ratios of immunoreactive positive cells of Col2, MMP3, MMP13 and ADAMTS5 in **(A) (C)** Immunohistochemistry results of iNOS, TNF-α and IL-6 proteins. Black arrows indicate a strong positive expression in cartilage tissue. **(D)** The ratios of immunoreactive positive cells of iNOS, TNF-α and IL-6 in **(C)**. Data are presented as mean ± SD (n = 6 mice per group). ^#^ *P* < 0.05, ^##^ *P* < 0.01, compared with the Sham group; *P < 0.05, **P < 0.01, compared with the ACLT + Vehicle group.

To further evaluate the protective effects of SR on cartilage matrix metabolism and inflammation *in vivo*, we examined the expression of key matrix and inflammatory markers in the articular cartilage of OA mice. Compared to the Sham group, the ACLT group exhibited a marked reduction in aggrecan and Col2 levels, accompanied by elevated expression of matrix-degrading enzymes MMP3, MMP13, and ADAMTS5. SR treatment effectively reversed these pathological changes (Figures 4A,B). Similarly, the expression of inflammatory mediators iNOS, TNF-α, IL-6, IL-1β and IL-18 in joint cartilage was significantly increased in the ACLT group, and this upregulation was markedly suppressed by SR administration (Figures 4C,D). Collectively, these results indicate that SR mitigates cartilage matrix degradation and local inflammation in OA mice, highlighting its therapeutic potential for OA management.

### 3.6 SR suppresses IL-1β–induced and OA-associated chondrocyte pyroptosis

**FIGURE 5:**
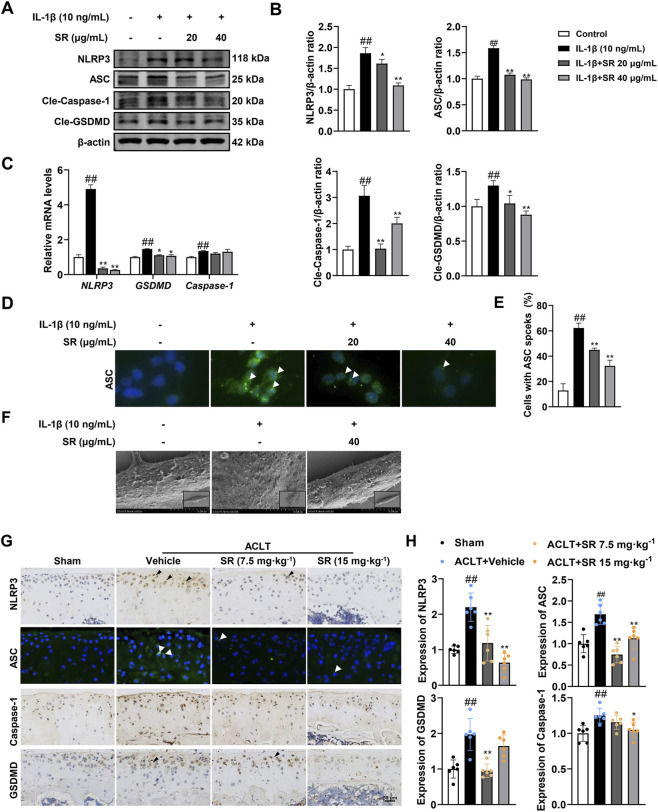
SR suppresses pyroptosis of chondrocytes. **(A-B)** The expression of NLRP3, ASC, Cle-Caspase-1, and Cle-GSDMD was visualized by Western blot and quantified using ImageJ. **(C)** The expression levels of pyroptosis-related genes were quantitatively analyzed by RT-qPCR method. **(D)** After IL-1β treatment with or without SR for 24 h, the formation of ASC specks was observed by immunofluorescence and the fluorescence intensity analyzed using ImageJ. DAPI stains nuclei blue. **(E)** Quantified percentage of cells containing an ASC speck. **(F)** Scanning electron microscopy showed that after IL-1β treatment, there were pyroptotic pores on the chondrocyte membrane, while after SR treatment, the formation of pyroptotic pores on the cartilage cell membrane decreased. **(G, H)** Immunohistochemistry staining results of NLRP3, ASC, Caspase-1, and GSDMD in articular cartilage 8 weeks post-ACLT surgery. Black arrows indicate a strong positive expression in cartilage tissue. Data are presented as mean ± SD. For **(A–E)**, n = 3 independent biological replicates; For **(G, H)**, n = 6 mice per group. ^#^ *P* < 0.05, ^##^ *P* < 0.01, compared with the Control group or Sham group; *P < 0.05, **P < 0.01, compared with the IL-1β group or the ACLT + Vehicle group.

Pyroptosis has been increasingly recognized as a critical driver of inflammation and cartilage degeneration in OA (Chang et al., 2022; An et al., 2020). And, our previous studies have demonstrated that upregulated chondrocyte pyroptosis play a key role in the progression of OA (Zhou et al., 2024; Yu et al., 2021a; Hu et al., 2020). Consistent with this, our data showed that SR treatment markedly reduced the levels of IL-1β and IL-18, key downstream effectors of pyroptosis, suggesting that SR may attenuate chondrocyte pyroptosis. To test this hypothesis, we first examined the activation of the NLRP3 inflammasome in IL-1β-stimulated chondrocytes. Western blot analysis revealed that IL-1β treatment significantly upregulated the expression of NLRP3, ASC, cleaved-Caspase-1, and cleaved-gasdermin D (GSDMD), whereas SR treatment markedly suppressed these increases (Figures 5A,B). In addition, RT-qPCR analysis revealed that SR suppressed the IL-1β-induced transcription of NLRP3, Caspase-1, and GSDMD (Figure 5C). Furthermore, IF staining revealed that SR treatment significantly reduced ASC speck formation in IL-1β-stimulated chondrocytes, indicating impaired macromolecular inflammasome complex assembly (Figures 5D,E).

To further validate the inhibitory effect of SR on pyroptosis at the cellular level, we assessed chondrocyte morphology using SEM analysis. The results showed that IL-1β treatment induced prominent membrane rupture—a hallmark characteristic of pyroptosis—while SR-treated cells exhibited largely intact membranes with only minimal pore formation (Figure 5F). These findings demonstrate that SR effectively suppresses NLRP3 inflammasome-mediated pyroptosis in chondrocytes *in vitro*.

We next investigated whether this antipyroptotic effect was maintained *in vivo*. IHC and IF analysis of articular cartilage revealed that OA mice exhibited markedly elevated expression of pyroptosis-associated proteins in chondrocytes, including NLRP3, ASC, Caspase-1, and GSDMD. Notably, SR treatment substantially reduced the expression of these markers (Figures 5G,H). Collectively, these results indicate that SR effectively mitigates chondrocyte pyroptosis both *in vitro* and *in vivo*, which may contribute to its overall chondroprotective effects in OA.

### 3.7 SR inhibits IL-1β and ACLT-induced NF-κB pathway activation in chondrocytes

**FIGURE 6:**
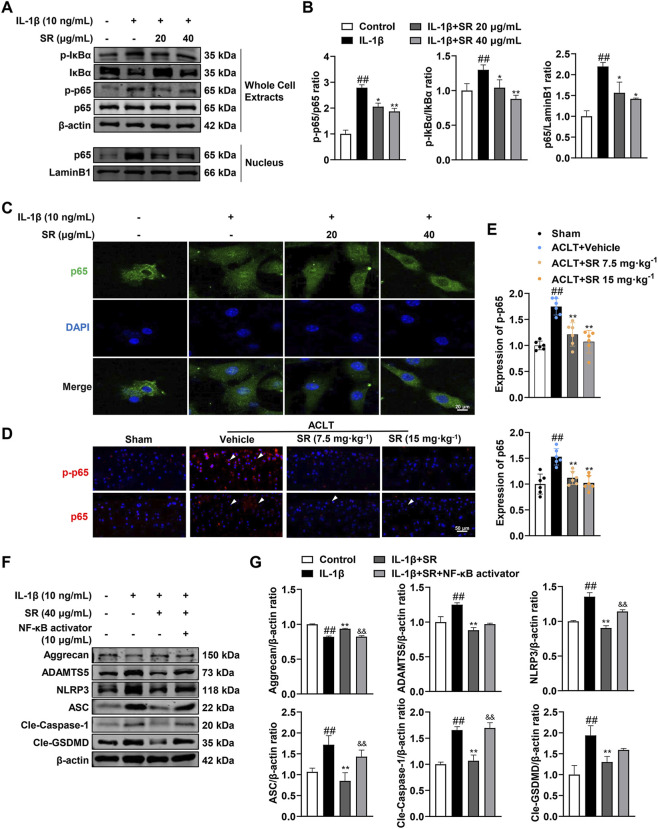
SR inhibits NF-κB activation in chondrocytes. **(A-B)** Western blot analysis showing the effect of SR on the expression and phosphorylation of IκBα and p65 in whole-cell lysates, and on nuclear p65 expression. **(C)** Immunofluorescence assay demonstrating the effect of SR on p65 nuclear translocation. DAPI stains nuclei blue. **(D)** Representative immunofluorescence staining of p-p65 and p65 in chondrocytes from the ACLT-induced OA model. DAPI stains nuclei blue. White arrows indicate p-p65-or p65-positive cells in cartilage tissue. **(E)** Quantitative analysis of p-p65 and p65 protein expression levels shown in panel **(D)**. **(F, G)** Western blotting results showing expression of Aggrecan, ADAMTS5, NLRP3, ASC, cleaved-Caspase-1, and cleaved-GSDMD in chondrocytes treated with NF-κB activator and subsequently treated with IL-1β in the presence or absence of SR. Data are presented as mean ± SD. For **(A-C, F, G)**, n = 3 independent biological replicates; For **(D, E)**, n = 6 mice per group. ^#^ *P* < 0.05, ^##^ *P* < 0.01, compared with the Control group or Sham group; *P < 0.05, **P < 0.01, compared with the IL-1β group or the ACLT + Vehicle group; ^and&^ *P* < 0.01, compared with the IL-1β + SR group.

To confirm whether the NF-κB pathway mediates the chondroprotective effects of SR, we assessed the expression and activation of key NF-κB pathway components—specifically IκBα and p65—in chondrocytes stimulated with IL-1β *in vitro*, as well as in chondrocytes within articular cartilage from OA mice. Western blot analysis demonstrated that IL-1β stimulation promoted the phosphorylation of IκBα and p65, triggered IκBα degradation, and facilitated nuclear translocation of p65. SR treatment effectively attenuated these pathological effects by reducing the phosphorylation of IκBα and p65, preserving cytoplasmic IκBα levels, and inhibiting p65 nuclear translocation (Figures 6A,B). These findings were further corroborated by IF staining, which revealed that SR markedly inhibited IL-1β-induced nuclear localization of p65 in chondrocytes (Figure 6C). Consistently, IF analysis of cartilage sections revealed that both total p65 and p-p65 were markedly elevated in chondrocytes of the ACLT group. Importantly, SR treatment significantly attenuated these elevations (Figures 6D,E). To validate whether the protective and anti-pyroptotic effects of SR are causally mediated by the inhibition of NF-κB signaling, we performed a functional rescue experiment utilizing a specific NF-κB activator. Western blot analysis showed that NF-κB activation markedly counteracted the protective effects of SR. In particular, the NF-κB activator attenuated SR-induced restoration of Aggrecan expression and reversed the SR-mediated downregulation of canonical inflammasome-related proteins, including NLRP3, ASC, and cleaved caspase-1 (Figures 6F,G). Collectively, these results indicate that SR suppresses chondrocyte pyroptosis and matrix degradation by inhibiting NF-κB signaling.

## 4 Discussion

**FIGURE 7:**
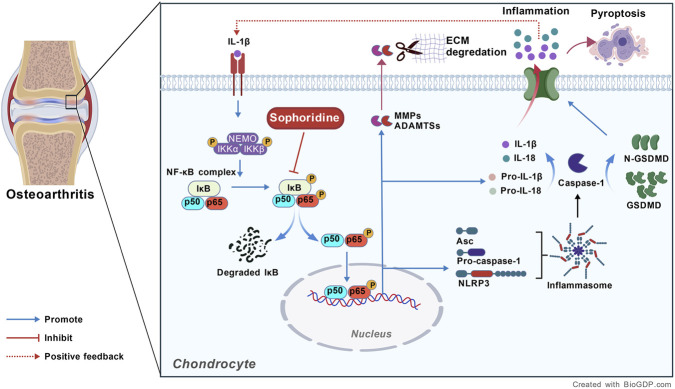
Schematic diagram of the protective effects of SR in OA. SR alleviates ECM degeneration and inflammation by suppressing NF-κB-driven activation of the NLRP3 inflammasome, thereby attenuating OA progression. Created with BioGDP.com.

Osteoarthritis (OA) represents the most prevalent degenerative joint disease worldwide, yet there are currently no approved disease-modifying therapies that effectively halt or reverse its progression (Chen et al., 2017; Lou et al., 2024). In light of this critical therapeutic gap, increasing attention has focused on bioactive compounds derived from traditional Chinese medicine as potential disease-modifying interventions (Chen et al., 2014). In the present study, we identified SR as a promising therapeutic candidate for OA treatment. *In vitro*, SR effectively inhibited ECM degradation, suppressed inflammatory responses, and reduced pyroptosis in IL-1β-stimulated chondrocytes. *In vivo*, SR administration significantly attenuated OA progression in an ACLT-induced mouse model, accompanied by decreased cartilage matrix degradation, reduced inflammation, and diminished chondrocyte pyroptosis. Mechanistically, these protective effects appear to be mediated, at least in part, through targeted inhibition of the NF-κB signaling pathway (Figure 7).

Chondrocyte protection is fundamental to preserving cartilage integrity and managing OA, as they represent the sole resident cells in articular cartilage and are responsible for synthesizing and maintaining the ECM, which is primarily composed of Col2 and aggrecan (Huang et al., 2025; De Roover et al., 2023). Under physiological conditions, chondrocytes maintain a balanced metabolic state; however, in OA and under inflammatory conditions, they adopt a pro-inflammatory phenotype and secrete pro-cytokines such as IL-1β, TNF-α, and IL-6. These mediators not only perpetuate local inflammation but also induce a detrimental phenotypic shift in chondrocytes toward a catabolic state, characterized by elevated expression of matrix-degrading enzymes such as MMPs and ADAMTSs, thereby accelerating ECM breakdown and cartilage degeneration (De Roover et al., 2023; Zheng et al., 2021a; Kim et al., 2015). In this study, we demonstrated that SR treatment preserved ECM homeostasis by maintaining Col2 and aggrecan expression, while concomitantly suppressing key catabolic enzymes such as MMP3, MMP13, and ADAMTS5 in chondrocytes both *in vitro* and *in vivo*. Furthermore, consistent with previous reports documenting SR’s anti-inflammatory effects (Wang et al., 2025; Zhang et al., 2008; Liang et al., 2022), SR markedly reduced the expression of inflammatory mediators in chondrocytes—including iNOS, COX-2, TNF-α, IL-6, IL-1β, and IL-18. Collectively, these findings highlight the potent chondroprotective and anti-inflammatory properties of SR in the context of OA.

A pivotal finding of our study is that SR effectively inhibited chondrocyte pyroptosis—an effect not previously reported—thereby revealing a novel mechanism underlying its chondroprotective and anti-inflammatory actions. Pyroptosis occurs via two primary pathways: the canonical and non-canonical pathways (Zheng et al., 2021b). Among these, the canonical pathway—primarily driven by NLRP3 inflammasome activation—has been most extensively studied in OA pathogenesis (Chen et al., 2024b). NLRP3 inflammasome activation typically proceeds through a two-step process: priming and activation. The priming step leads to increased expression of inflammasome components, including NLRP3, pro-IL-1β and pro-18. The activation step involves assembly of the inflammasome complex, Caspase-1 activation, cleavage of GSDMD, and maturation of IL-1β and IL-18, ultimately leading to pore formation and pro-inflammatory cytokine release (Swanson et al., 2019). Our data demonstrated that SR treatment significantly suppressed chondrocyte pyroptosis in response to IL-1β stimulation, as evidenced by reduced expression of NLRP3, ASC, cleaved-Caspase-1, cleaved-GSDMD, and the mature forms of IL-1β and IL-18, as well as the ASC speck formation. Notably, SR also downregulated the mRNA levels of these key pyroptotic markers. Consistent with the *in vitro* findings, SR treatment reduced the protein expression of these markers in chondrocytes from OA mice. These results suggest that SR predominantly interferes with the priming phase of NLRP3 inflammasome-mediated pyroptosis in chondrocytes during OA progression.

NF-κB signaling serves as a well-established upstream regulator of the priming phase of NLRP3 inflammasome activation (Swanson et al., 2019), and simultaneously plays a central role in OA pathogenesis (Liu et al., 2017; Choi et al., 2019). In OA chondrocytes, NF-κB can be activated by mechanical stress, pro-inflammatory cytokines such as IL-1β and TNF-α, and fibronectin fragments, subsequently promoting the expression of catabolic enzymes and pro-inflammatory mediators that accelerate cartilage degradation. Under resting conditions, NF-κB dimers (typically p50/p65 heterodimers) are sequestered in the cytoplasm by IκBα. Upon stimulation, IκBα undergoes phosphorylation and subsequent degradation via the proteasome, thereby allowing phosphorylated p65 to translocate into the nucleus and initiate transcription of pro-inflammatory genes (Fang et al., 2024). Consistent with this established mechanism, our data revealed that SR robustly inhibited NF-κB pathway activation in IL-1β-stimulated chondrocytes. SR treatment suppressed the phosphorylation of IκBα and p65, prevented IκBα degradation, and reduced nuclear translocation of p65. *In vivo*, SR administration also attenuated the phosphorylation of IκBα and p65 in OA cartilage. Furthermore, an NF-κB activator significantly counteracts the therapeutic effects of SR. These findings are consistent with previous reports demonstrating that SR suppresses NF-κB signaling to exert anti-inflammatory effects (Chen et al., 2024a; Liang et al., 2022; Wang et al., 2025).

Recently, a study reported that SR exerts therapeutic effects in rheumatoid arthritis (RA), a pathologically distinct form of arthritis characterized by autoimmune-driven synovial inflammation. In a CIA rat model, SR treatment significantly reduced synovial inflammation and bone erosion (Chen et al., 2024a). Given that FLSs are key mediators of RA pathogenesis, the study further examined the effects of SR on TNFα-stimulated arthritic FLSs and demonstrated that SR effectively inhibited their proliferation and migration. Notably, FLSs are also known to contribute to synovial inflammation and disease progression in OA (Han et al., 2020). Thus, while our study did not directly assess SR’s effects on FLSs, these findings help address that limitation and, together, support the multi-target therapeutic potential of SR in joint diseases.

Beyond therapeutic efficacy, safety is a critical consideration for clinical translation. In our study, SR was administered intraperitoneally at doses of 7.5 and 15 mg/kg, both of which significantly alleviated OA symptoms and cartilage degradation, with the higher dose yielding more pronounced effects. When converted to human-equivalent doses using established allometric scaling methods, these correspond to approximately 0.53 and 1.07 mg/kg, respectively (Nair and Jacob, 2016). Importantly, these doses are substantially lower than the clinically approved dose of SR for treating malignant trophoblastic tumors (125 mg/m^2^, equivalent to ∼3.38 mg/kg in humans) (Tang et al., 2022), suggesting that the doses used in our study fall well within a safe range. To further evaluate the safety profile of SR, we monitored body weight and conducted histopathological assessments of the liver and kidneys. SR administration did not induce any observable toxic effects in these organs (Supplementary Figure S1), further reinforcing its favorable safety profile. Together, SR emerges as a promising candidate for OA treatment, offering both therapeutic efficacy and a strong safety margin to support future clinical investigation.

Despite these promising findings, several limitations of this study should be acknowledged. First, although *in vitro* rescue data demonstrated that SR suppresses pyroptosis and matrix degradation by inhibiting NF-κB, we did not perform *in vivo* pathway interventions. Future studies using gene-targeting animal models are needed to definitively validate SR’s therapeutic mechanism *in vivo*. Second, we did not investigate whether SR may exert its protective effects in OA through additional signaling pathways or alternative mechanisms beyond NF-κB inhibition. Third, the direct molecular target of SR in chondrocytes remains unidentified, and the precise mechanism by which SR downregulates NF-κB signaling remains unclear. Fourth, OA is a multifactorial disease involving not only chondrocytes but also other key cell types, such as fibroblast-like synoviocytes (FLSs) and synovial macrophages (Liu et al., 2024a), which were not evaluated in the present study. Fifth, we only assessed the efficacy of SR via intraperitoneal injection; we did not explore alternative administration routes, particularly intra-articular injection, which may offer advantages by minimizing systemic side effects and enhancing local drug concentrations to improve therapeutic efficacy (Kuang et al., 2025). Future research should therefore include more comprehensive investigations to fully elucidate the complete therapeutic potential and mechanisms of SR in OA. In addition, the development of SR-loaded delivery systems using nanomaterials or other biomaterials—which have shown significant promise in enhancing drug bioavailability, stability, and targeted delivery (Peng et al., 2024; Kalairaj et al., 2024; Yang et al., 2025)—may represent a valuable strategy to optimize its clinical application in OA treatment.

## 5 Conclusion

In conclusion, SR effectively preserved ECM homeostasis and suppressed inflammatory responses in OA chondrocytes, leading to a marked attenuation of OA progression. These protective effects are associated with the inhibition of NF-κB/NLRP3 signaling-driven inflammatory and pyroptotic pathways. Collectively, these findings strongly highlight the promising therapeutic potential of SR for the treatment of OA.

## Data Availability

The original contributions presented in the study are included in the article/Supplementary Material, further inquiries can be directed to the corresponding authors.
