# Supplementary material for: Sophoridine attenuates osteoarthritis progression: association with suppression of chondrocyte pyroptosis via inhibiting NF-κB signaling pathway
Source: Front Pharmacol. 2026 Jul 15;17:1861567. doi: 10.3389/fphar.2026.1861567 (PMC13414949; doi:10.3389/fphar.2026.1861567)

Brand: Epizyme Biomedical
Product Name: Three-color Prestained Protein Marker
Catalogue Number: Cat# WJ103

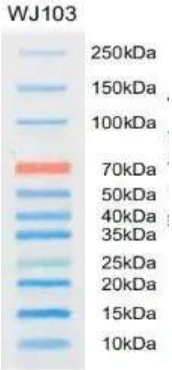

Figure5 (A)

ASC+GAPDH

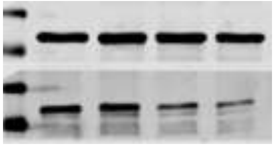

Figure6 (G)

Aggrecan+β-actin

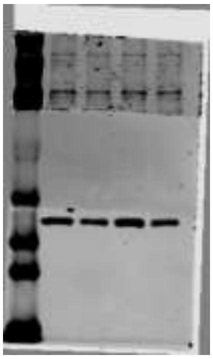

ADAMTS5+β-actin

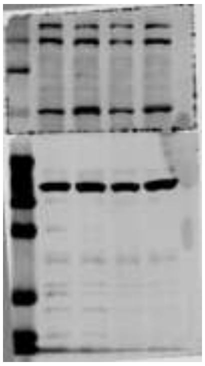

NLRP3+β-actin

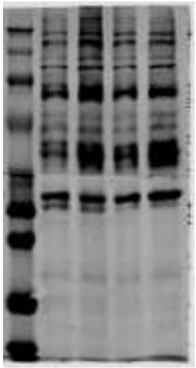

ASC+β-actin

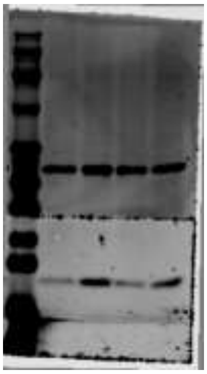

Cle-caspase1+β-actin

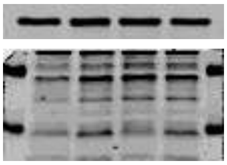

Cle-GSDMD+β-actin

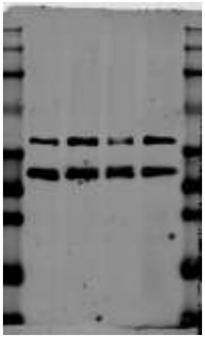

Supplement: Supplementary file 1 [file DataSheet2.pdf]
