# Supplementary material for: Sophoridine attenuates osteoarthritis progression: association with suppression of chondrocyte pyroptosis via inhibiting NF-κB signaling pathway
Source: Front Pharmacol. 2026 Jul 15;17:1861567. doi: 10.3389/fphar.2026.1861567 (PMC13414949; doi:10.3389/fphar.2026.1861567)
Supplement: Supplementary file 2 [file Supplementaryfile1.docx]

Supplementary Material


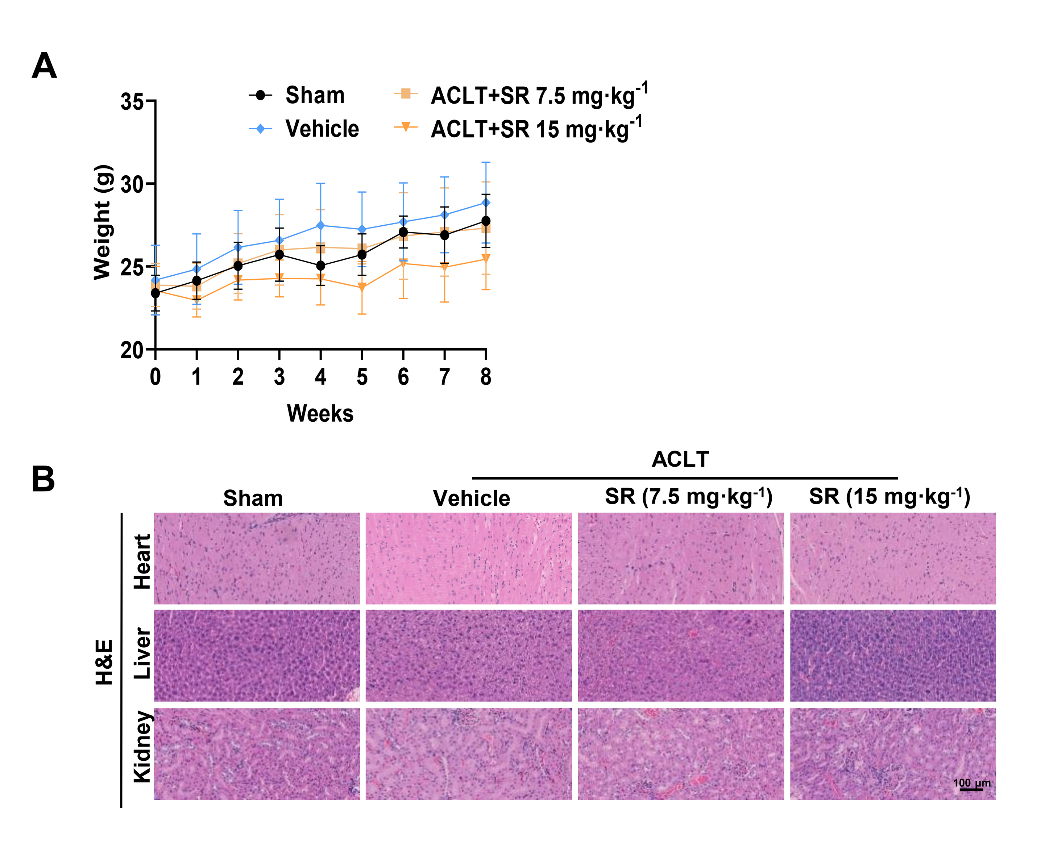


**Supplementary Figure 1. SR exhibits favorable systemic tolerance with no evident toxicity in major organs.** **(A)** Weekly postoperative body weight changes in mice across different treatment groups over 8 weeks. **(B)** Representative H&E-stained sections of the heart, liver, and kidney from each group.

**Supplementary Table S1. Summary of one-way ANOVA parameters and post-hoc power analysis for primary outcome measures.**

| **Outcome Measure** | **F-statistic (F)** | **Degrees of Freedom (df₁, df₂)** | ***P*-value** | **R^2^** | **Cohen’s f (Effect Size)** | | **Achieved Power (α = 0.05)** |
| --- | --- | --- | --- | --- | --- | --- | --- |
| BV/TV (%) | 34.06 | （3, 20） | <0.0001 | 0.8363 | 2.2603 | 1.0000 | |
| Tb.Th (mm) | 6.336 | （3, 20） | 0.0034 | 0.4873 | 0.9749 | 0.9651 | |
| Tb.Sp (mm) | 9.023 | （3, 20） | 0.0006 | 0.5751 | 1.1634 | 0.9955 | |
| Mechanical threshold (g) | 5.265 | （3, 20） | 0.0077 | 0.4413 | 0.8887 | 0.9264 | |
| Paw withdrawal threshold (s) | 6.395 | （3, 20） | 0.0032 | 0.4896 | 0.9794 | 0.9665 | |
| Ratio (RH/LH): Swing | 6.378 | （3, 20） | 0.0033 | 0.4890 | 0.9781 | 0.9661 | |
| Ratio (RH/LH): Stride length | 6.142 | （3, 20） | 0.0039 | 0.4795 | 0.9598 | 0.9599 | |
| Ratio (RH/LH): Paw area | 11.44 | （3, 20） | 0.0001 | 0.6318 | 1.3100 | 0.9994 | |
| OARSI Scores | 26.45 | （3, 20） | <0.0001 | 0.7987 | 1.9919 | 1.0000 | |
| Expression of Aggrecan | 29.59 | （3, 20） | <0.0001 | 0.8161 | 2.1068 | 1.0000 | |
| Expression of Col2 | 7.046 | （3, 20） | 0.002 | 0.5138 | 1.0281 | 0.9792 | |
| Expression of MMP3 | 9.213 | （3, 20） | 0.0005 | 0.5802 | 1.1756 | 0.9961 | |
| Expression of MMP13 | 14.95 | （3, 20） | <0.0001 | 0.6917 | 1.4975 | 1.0000 | |
| Expression of ADAMTS5 | 4.848 | （3, 20） | 0.0108 | 0.4210 | 0.8528 | 0.9029 | |
| Expression of iNOS | 11.39 | （3, 20） | 0.0001 | 0.6309 | 1.3071 | 0.9994 | |
| Expression of TNF-α | 4.764 | （3, 20） | 0.0115 | 0.4168 | 0.8453 | 0.8974 | |
| Expression of IL-6 | 18.48 | （3, 20） | <0.0001 | 0.7349 | 1.6649 | 1.0000 | |
| Expression of IL-1β | 18.07 | （3, 20） | <0.0001 | 0.7304 | 1.6464 | 1.0000 | |
| Expression of IL-18 | 16.89 | （3, 20） | <0.0001 | 0.7169 | 1.5917 | 1.0000 | |
| Expression of NLRP3 | 21.77 | （3, 20） | <0.0001 | 0.7656 | 1.8071 | 1.0000 | |
| Expression of ASC | 18.52 | （3, 20） | <0.0001 | 0.7353 | 1.6667 | 1.0000 | |
| Expression of Caspase-1 | 6.594 | （3, 20） | 0.0028 | 0.4973 | 0.9945 | 0.9710 | |
| Expression of GSDMD | 14.95 | （3, 20） | <0.0001 | 0.6917 | 1.4975 | 1.0000 | |
| Expression of p-p65 | 20.99 | （3, 20） | <0.0001 | 0.759 | 1.7744 | 1.0000 | |
| Expression of p65 | 14.53 | （3, 20） | <0.0001 | 0.6854 | 1.4763 | 1.0000 | |
