# Supplementary material for: Sophoridine attenuates osteoarthritis progression: association with suppression of chondrocyte pyroptosis via inhibiting NF-κB signaling pathway
Source: Front Pharmacol. 2026 Jul 15;17:1861567. doi: 10.3389/fphar.2026.1861567 (PMC13414949; doi:10.3389/fphar.2026.1861567)

Brand: Epizyme Biomedical

Product Name: Three-color Prestained Protein Marker

Catalogue Number: Cat# WJ103

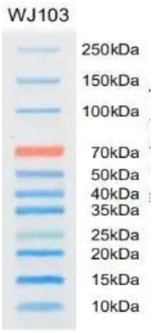

Figure1 (e)

WJ103, Epizyme Biomedical Technology Co., Ltd, Shanghai, China

(Aggrecan(150kDa), Col2 (140kDa), mmp3 (54kDa), mmp13 (60kDa), adamts5 (73kDa), β-actin (42kDa), gapdh (37kDa)

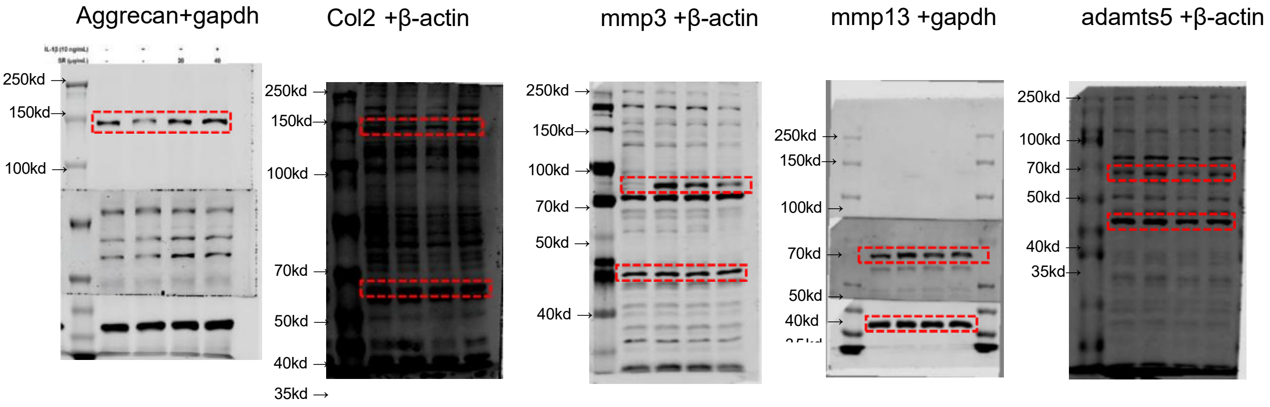

Figure2 (a)

WJ103, Epizyme Biomedical Technology Co., Ltd, Shanghai, China

(iNOS (130kDa), COX2 (68kDa), TNF-α (23kDa), IL-6 (21kDa), IL-1β (17kDa), IL-18 (22kDa) ,β-actin (42kDa)

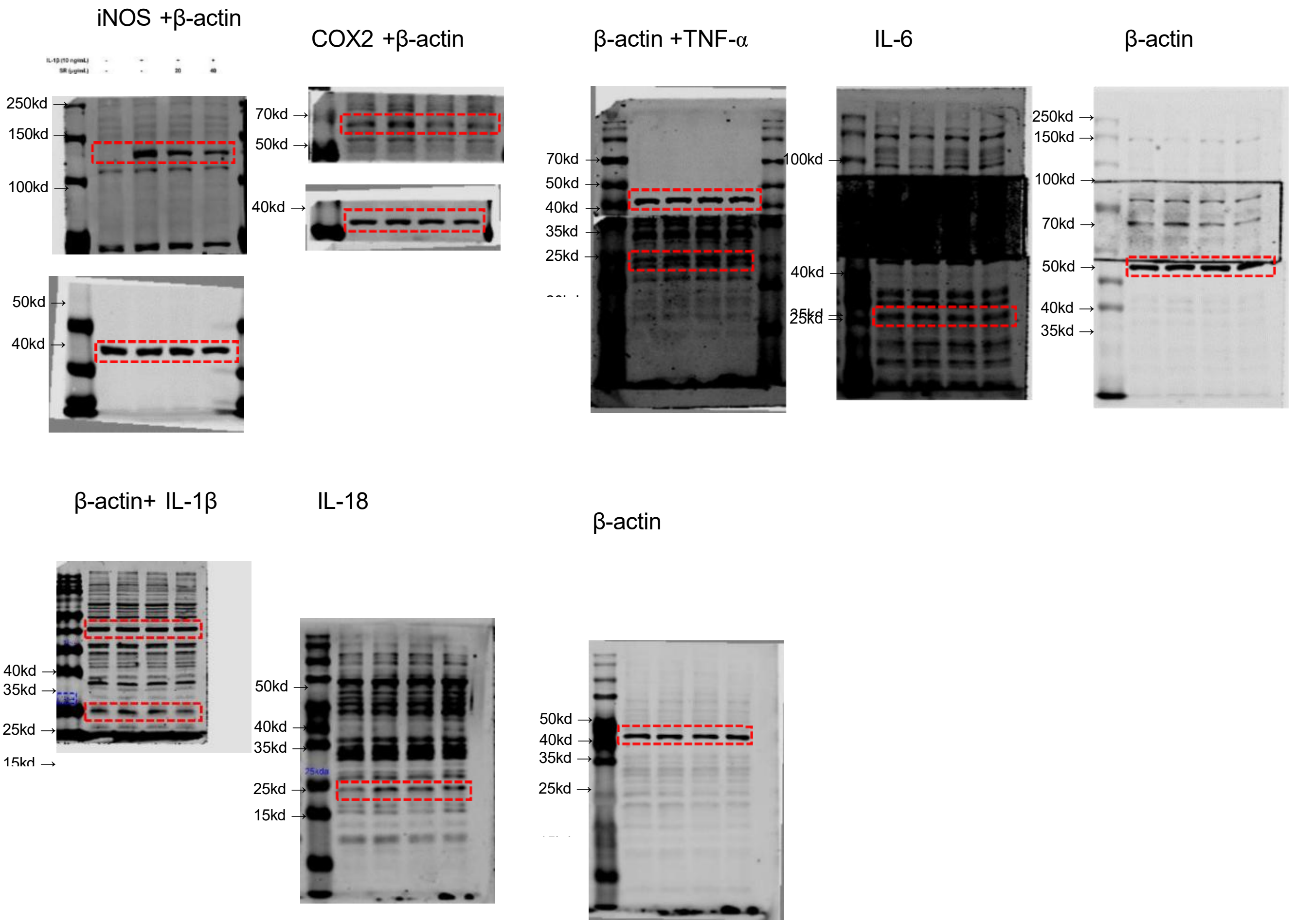

Figure5 (a)

WJ103, Epizyme Biomedical Technology Co., Ltd, Shanghai, China

(NLRP3 (118kDa), Cle-CASPASE1 (20kDa), Cle-GSDMD (35kDa),β-actin (42kDa)

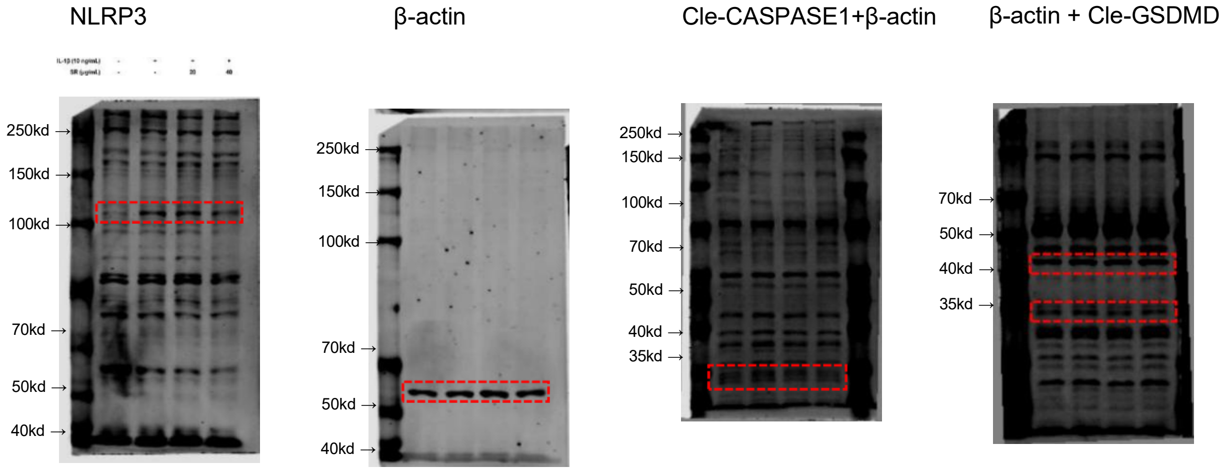

Figure6 (a)

WJ103, Epizyme Biomedical Technology Co., Ltd, Shanghai, China

(p-iκBα (35kDa), iκBα (35kDa), P65 (65kDa), p-P65(65kDa),β-actin (42kDa), LaminB1(66kDa)

Whole Cell Extracts

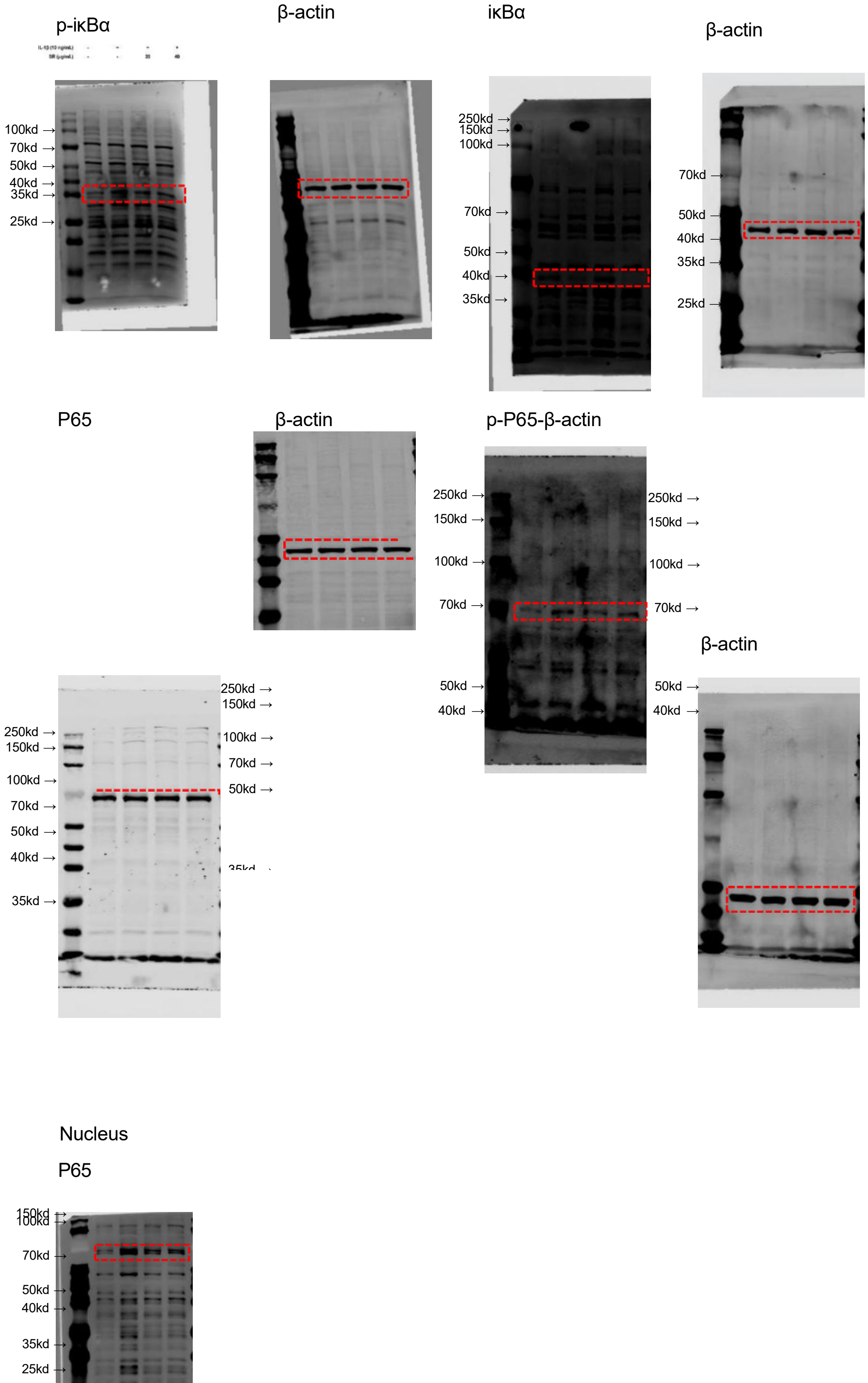

LaminB1

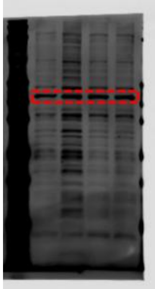

Supplement: Supplementary file 3 [file DataSheet1.pdf]
